# Infrapopliteal Artery Occlusive Disease: An Overview of Vessel Preparation and Treatment Options

**DOI:** 10.3390/jcm9103321

**Published:** 2020-10-16

**Authors:** Srini Tummala, Ayush Amin, Ankit Mehta

**Affiliations:** Department of Interventional Radiology, University of Miami Health System, Miami, FL 33136, USA; Ayush.amin@jhsmiami.org (A.A.); Ankit.mehta@jhsmiami.org (A.M.)

**Keywords:** critical limb ischemia, infrapopliteal artery disease, atherectomy, vessel preparation, review, tibial disease, BTK disease, peripheral artery disease

## Abstract

Critical limb ischemia (CLI) is defined as chronic rest pain and/or the presence of tissue loss (ulcers or gangrene) in the lower extremities secondary to ischemia. CLI is a limb and potentially life-threatening disease associated with a poor prognosis with only 50% of patients being able to preserve both limbs within 12 months of diagnosis. CLI related to diabetes is often more extensive with multi-level long segmental arterial disease resulting in a 5–30-fold increased rate of amputation. As the incidence and prevalence of diabetes mellitus increases within our aging society, the rate of infrapopliteal artery occlusive disease (IPOD) and the need for intervention rises with it. The aim of this manuscript is to provide the reader with an overview of the various devices available for vessel preparation (VP) and treatment of IPOD in order to optimize patency rates, symptom resolution, healing of wounds, and minimize complications.

## 1. Introduction

Critical limb ischemia (CLI) is defined as chronic rest pain and/or the presence of tissue loss (ulcers or gangrene) in the lower extremities secondary to ischemia. CLI is a limb and potentially life-threatening disease and represents end-stage peripheral arterial disease (PAD), occurring in about 10% of PAD patients. It is associated with a poor prognosis with only 50% of patients being able to preserve both limbs within 12 months of diagnosis [1,2,3].

A large percentage of CLI patients have infrapopliteal arterial occlusive disease (IPOD) especially within the diabetic population where PAD is 3–4 times more common. CLI related to diabetes is often more extensive with multi-level long segmental arterial disease resulting in a 5–30-fold increased rate of amputation [4,5,6,7]. As the incidence and prevalence of diabetes mellitus increases within our aging society, the rate of IPOD and the need for intervention rises with it.

The presence of various underlying co-morbidities and lack of autogenous venous conduit renders a significant number of CLI patients unsuitable for bypass surgery. Endovascular treatment (EVT) has been shown to have less morbidity and mortality compared to open surgery, while being equivalent in efficacy [8,9,10,11,12,13]. Given these advantages, EVT of IPOD has become a first-line therapy [14,15,16,17,18,19]. Whether its EVT or open surgery, the goal of treatment is to relieve the ischemic pain, promote wound healing, salvage the limb, improve the patients’ functionality, improve quality of life, and gain amputation-free survival [4].

In general, infrapopliteal artery (IPA) interventions are technically challenging due to the presence of chronic total occlusions (CTOs), defined as 100% arterial occlusion lasting more than 30 days. CTOs in the infrapopliteal region are often diffuse, extensive and can be found in up to 55% of patients [6,20]. CTOs are typically composed of extensive calcium deposits that make successful crossing challenging, with a failure rate of 20% or more depending on the operators’ experience. IPA CTOs can result in prolonged procedure time, increased radiation exposure, higher contrast volume, higher chance for dissection, and increased likelihood of arteriovenous fistula formation and arterial perforation. In addition, there are limited bailout options when dealing with below the knee (BTK) complications. As a result, those treating patients with CLI and IPOD must be proficient in the various devices available for vessel preparation and treatment in order to minimize complications and optimize patency rates, symptom resolution, and healing of wounds.

### 1.1. Vessel Preparation (VP)

Vessel preparation (VP) is a crucial component of EVT. VP minimizes the risk of dissection and rupture, maximizes luminal gain, and prepares the vessel for local drug delivery, vascular mimetic implants, or stents. It should be considered regardless of whether the lesion is stenotic or occlusive and is especially crucial if calcium is present. In severely calcified arteries, dissections, suboptimal luminal gain, and local drug delivery is not ideal due to the reduced compliance of the artery and the mechanical barrier (calcification) in the arterial wall to local drug delivery. These challenges can be overcome by dedicated VP including undersized balloons, cutting balloons, scoring balloons, and atherectomy [21]. Proper VP affects the procedural success rate and long-term outcomes. Consequently, VP has shifted from a trend to a consistent element of treatment algorithms.

### 1.2. Cutting Balloon/Scoring Balloon

**Figure 1:**
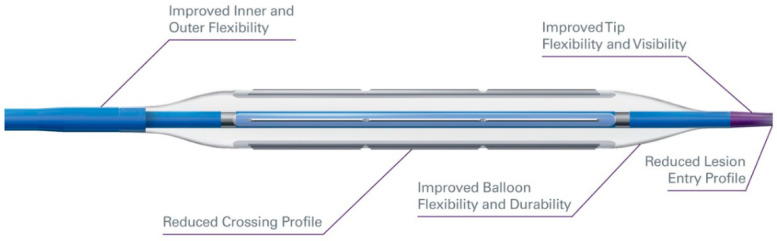
Wolverine Cutting Balloon. Courtesy Boston Scientific Corporation.

**Figure 2:**
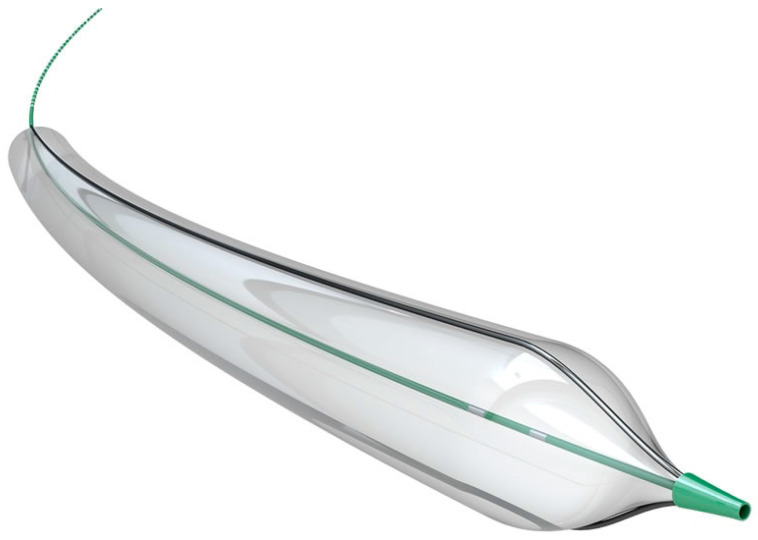
Ultrascore Focused Forced percutaneous transluminal angioplasty (PTA) (Scoring Balloon). Courtesy of BD BARD Peripheral Vascular.

Cutting balloons and scoring balloons essentially combine the features of a conventional balloon with atherotomes or wires mounted on the surface, respectively. These atherotomes or wires function as microsurgical blades that give these balloons their cutting or scoring properties (Figure 1 and Figure 2).

They were originally designed to treat restenosis of coronary stents secondary to neo-intimal hyperplasia [22,23]. However, their utility has expanded to include PAD and specifically IPOD given that these devices are well suited for treating fibrous and calcified lesions that are resistant to dilation. The rationale behind this technology is that the entire force is focused on a wire or blade edge mounted on the balloon. This leads to a controlled plaque incision or a controlled dissection with less barotrauma to the entire lesion, thus reducing the extent of dissection, lowering the risk of rupture, minimizing plaque shift, decreasing vessel elastic recoil, and potentially reducing the inflammatory response. Since the degree of vascular injury and dissection is known to be a correlator of restenosis, there is a significant potential in improved outcomes [24]. Balloons in this category include the Wolverine (Boston Scientific, Maple Grove, MN, USA), Ultrascore (BD Peripheral Vascular Inc., Tempe, AZ, USA), and the Angiosculpt (Royal Philips, Amsterdam, The Netherlands).

Although none of these cutting and scoring balloons have an indication for IPOD, several studies have shown the benefits of them for VP. Ansel et al. showed an overall limb salvage rate of 89.5% at 1 year using cutting balloons to treat IPOD [24]. Lezzi et al. showed the effectiveness of cutting balloons to treat IPA bifurcation disease with a technical success of 100%, no reported 30-day mortality, and a 1 year primary and secondary patency rate of 74.2% and 87.0%, respectively [25]. Finally, Canaud et al. reported an overall technical success of 96.3% and a complication rate of 8.9% treating de novo infra-inguinal arterial lesions [26].

### 1.3. Atherectomy

Another common method for VP is atherectomy which results in plaque modification due to sanding, drilling/aspiration, removing, and vaporizing. Currently 4 main types of atherectomy are available including: orbital, rotational, directional, and laser.

### 1.4. Orbital Atherectomy (OA)

**Figure 3:**
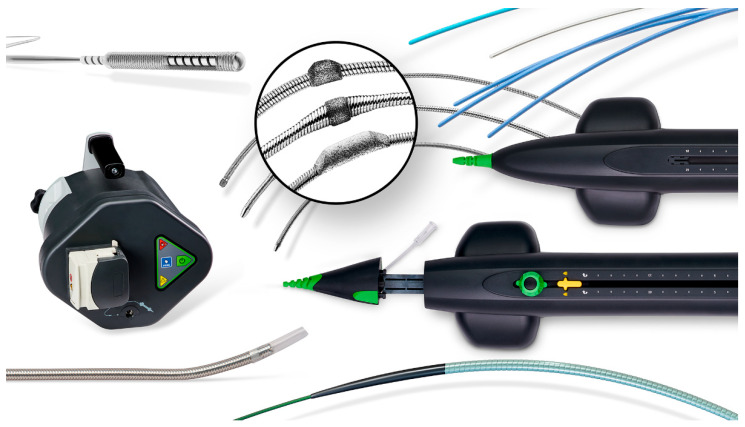
CSI Diamondback 360 Peripheral Orbital Atherectomy System. Courtesy of Cardiovascular Systems, Inc.

Orbital atherectomy (OA) using the Diamondback 360 degree Peripheral Orbital Atherectomy System (Cardiovascular Systems Inc., St. Paul, MN, USA) uses centrifugal force and bi-directional sanding which differentially sands away arterial calcium from atherosclerotic tissue to form smooth and larger lumens without damaging compliant arterial tissue [27] (Figure 3).

Final lumen diameter is dependent on the selected crown size and the rotational speed (80 K to 200 K rpm) during the atherectomy. A unique feature of OA is the mechanism of operation in which the luminal diameter increases proportionally to crown rotational speed and crown weight. This circumferential removal of calcium has been hypothesized to improve vessel compliance and facilitate low-pressure angioplasty and reduce dissections and rupture [28].

Several studies have shown the safety and effectiveness of OA with IPOD. The Orbital Atherectomy System for the Treatment of Peripheral Vascular Stenosis (OASIS) trial is a nonrandomized, investigational device exemption study. It was the first major study to investigate the safety and efficacy of OA in IPOD. This study showed a high procedural success rate of 90.1%, defined as a final diameter stenosis of <30%, and a low rate of major adverse events of 10.4% at 6 months. No patients had major amputations and ankle brachial index (ABI) scores remained significantly improved (*p* < 0.0001) at 6 months [28]. The Calcium 360 study is a randomized multicenter study comparing OA with adjunctive percutaneous transluminal angioplasty (PTA) versus PTA alone in patients with severely calcified IPOD. Procedural success was 93.1% for OA plus PTA versus 82.4% for PTA. Bailout stenting was needed in 6.9% of the OA plus PTA treated lesions and 14.3% of the PTA treated lesions. At 1 year, there were no amputations in either group related to the index procedure. Estimates for freedom from target lesion revascularization (TLR) and all-cause mortality were 93.3% and 100% in the OA plus PTA group versus 80.0% in the PTA only group, respectively. Debulking with OA appeared to increase the chance of reaching a desirable angioplasty result, with less acute need for bailout stenting and a higher procedure success [29].

The CONFIRM I, II, and III registries are multicenter, non-randomized, all-comer registries of all patients with PAD who were treated with OA in the United States. A total of 1842 diabetic patients (1111 men; mean age 70.6 ± 10.2 years) with 2819 lesions and 1247 non-diabetic patients (732 men; mean age 72.9 ± 10.7 years) with 1885 lesions were included. There were no inclusion or exclusion criteria for enrollment.

In a sub-analysis based on lesion location of the CONFIRM registry patients, there was an overall procedural success of 93.1% in patients treated with OA and PTA versus 82.4% for PTA alone. Patients with above the knee (ATK) and popliteal artery lesions had a higher final residual stenosis (10% vs. 9%; *p* = 0.004) and required the use of more adjunctive therapies (e.g., balloons and stents; 1.3% vs. 1.1%; *p* < 0.001) compared to patients with BTK disease. Patients with IPOD also had a higher incidence of perforation (1.5% vs. 0.2%; *p* = 0.005), slow flow (7.7% vs. 5.0%; *p* = 0.03) and spasm (10.3% vs. 4.2%; *p* < 0.001) but a lower incidence of distal embolization (0.4% vs. 5.1%; *p* < 0.001) [30].

In the most recent study, which extracted data from the LIBERTY 360 registry adjunctive OA was studied for 617 lesions, 54.1% of which were solely BTK. Among these lesions 108 were Rutherford category (RC) 2–3, 174 were RC 4–5, and 52 were RC 6. Among these Critical Limb Threatening Ischemia (CLTI) patients, survival rates were 76.2% for RC 4–5 and 63.7% for RC 6 and there was a low amputation rate (4.7% and 11.4%, respectively). These results suggest that OA with adjunctive PTA in patients with CLI is safe and effective across RC groups and is associated with a low major amputation rate after 3 years of follow-up [29].

### 1.5. Rotational Atherectomy (RA)

**Figure 4:**
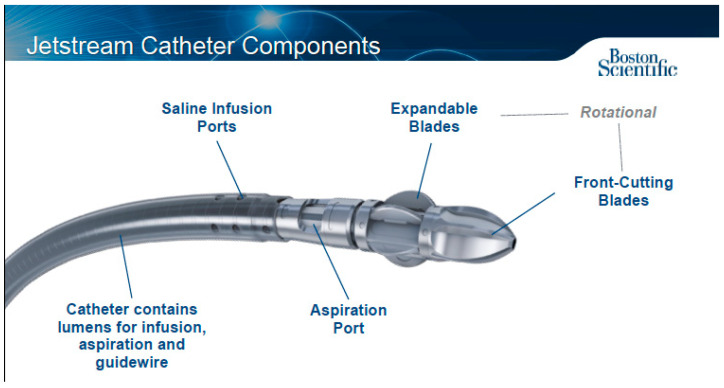
Jetstream Atherectomy System. Courtesy Boston Scientific Corporation.

**Figure 5:**
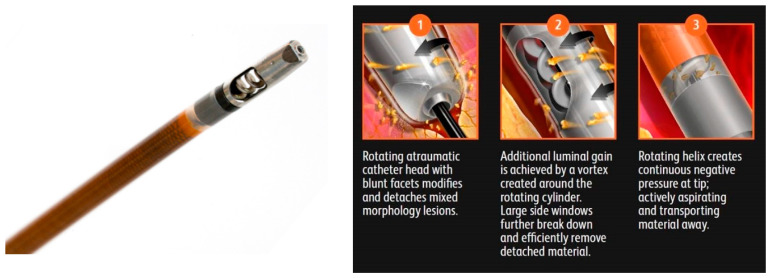
Rotarex Rotational Excisional Atherectomy System. Image 1 shows rotating atraumatic catheter head which modifies plaque and detaches mixed morphology lesions. Image 2 shows additional luminal gain achieved by vortex created around the rotating cylinder. Side windows break down and remove detached material. Image 3 shows rotating helix which creates continuous negative pressure at tip to actively aspirate and transport material away. Courtesy of BD BARD Peripheral Vascular.

Rotational atherectomy (RA) is another VP technique where atherosclerotic plaque is shaved by a centrally rotating cutter known as a tip or a burr. Oftentimes these devices have an associated aspiration capability which allows them to treat all morphologies including soft plaque, calcification, and thrombus. With these devices the luminal gain is directly proportional to the size of the cutter used [27]. Devices in this category include the Jetstream (Boston Scientific Corporation, MN, USA) (Figure 4), Rotablator (Boston Scientific), Phoenix (Philips), and the Rotarex (BD Peripheral Vascular Inc.) (Figure 5).

Overall, there is limited high quality data regarding the use of RA for IPOD. The earliest known study was performed in 1991 by Dorros et al. who studied the clinical outcomes of RA using the Rotablator (Boston Scientific, MN). In this study, they achieved an overall device technical success of 95%, which included 37 IPA lesions [31]. In 2009, Zeller et al. treated 18 IPA lesions with Jetstream (Boston Scientific, MN). Device technical success was 99% with a 1% major adverse event rate at 30 days. The one-year restenosis rate for IPA disease was 11.1% based on duplex arterial imaging [32]. In 2011, Sixt et al. described their one-year outcomes after RA with Jetstream (Boston Scientific, MN) in infra-inguinal arteries, of which they treated 16 IPA lesions, with an overall device success of 99% [33].

### 1.6. Directional Atherectomy (DA)

**Figure 6:**
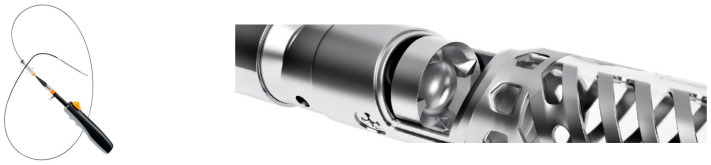
HawkOne^TM^ Directional Atherectomy System. ©2020 Medtronic. All rights reserved. Used with the permission of Medtronic.

Directional atherectomy (DA) removes plaque by guiding a motorized cutter of the catheter towards the plaque itself and then advancing or rotating it to remove plaque and capture it in its nosecone (Figure 6).

**Figure 7:**
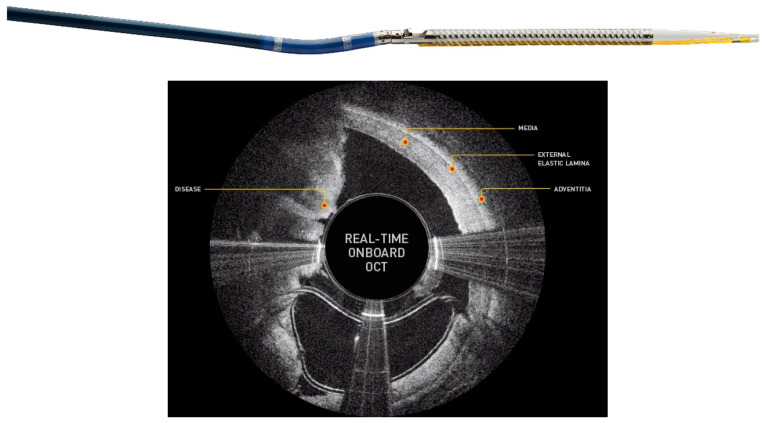
Pantheris System with Onboard OCT Guided Atherectomy. Courtesy of Avinger, Inc.

This makes it especially well suited for treating eccentric lesions. Since there is no aspiration system the nose cone must be intermittently emptied via catheter retrieval prior to further debulking to reduce the chance of distal embolization [34]. Devices included in this category are Medtronic’s SilverHawk (Minneapolis, MN, USA), TurboHawk, and their most recent product, the HawkOne. The HawkOne claims to provide twice the cutting efficiency of the Turbohawk and simplifies the cleaning process through a preloaded distal flush tool. Another relatively new DA is the Pantheris optical coherence tomography (OCT) guided atherectomy device (Avinger Inc., Redwood City, CA, USA) (Figure 7).

With its incorporation of OCT, it is possible to simultaneously visualize and remove plaque, making this catheter well suited for targeting eccentric plaque and minimizing cutting into the adventitia or even perforating an artery [35].

Regarding IPOD, there are no RCTs related to DA. However, multiple studies describing the utility of SilverHawk in treating IPA lesions have been reported. In 2006, Ramaiah et al. published their results using the SilverHawk device from their multi-institutional TALON registry, which included 601 patients and 1258 lesions, of which 317 were infrapopliteal. Unfortunately, they did not discuss their results specifically for IPOD. However, their overall procedural success, defined as <50% residual diameter stenosis, was 97.6%, with a 1-year target TLR rate of 20% [36]. In 2007, Zeller et al. treated 49 IPA lesions with SilverHawk, defined as <30% residual stenosis, with a 98% procedural success. They also described primary and secondary patency rates of 67% and 91% after 1 year and 60% and 80% after 24 months [37].

Finally, in the more recent prospective, multicenter DEFINITIVE LE trial, 189 IPA lesions were treated with SilverHawk. Rastan et al. described a success rate of 84% (defined as </= 30% residual stenosis), with a 1-year overall primary patency rate of 84% [38].

### 1.7. Laser Atherectomy (LA)

**Figure 8:**
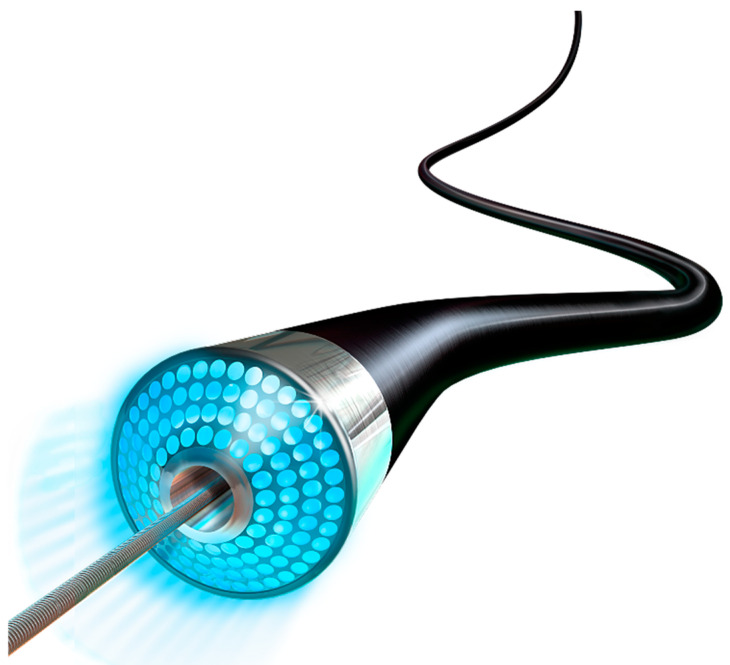
Turbo-Elite Laser Atherectomy System. Courtesy of Philips USA.

Laser atherectomy (LA) is an excimer laser which uses ultraviolet radiation to vaporize and remove plaque and thrombus from the arterial lumen (Figure 8).

The benefit of ablating tissue using this photochemical process as opposed to thermally is that there is no rise in the temperature of the surrounding non-target tissue. Furthermore, the laser functions with a lower penetration depth to avoid damaging nearby tissue [27,34]. Devices included in this category are the Turbo-Tandem, Turbo-Elite, Turbo-Power (Spectranetics Corporation, Colorado Springs, CO, USA).

In 2002, Gray et al. prospectively evaluated the use of LA in CLI patients, of which 32% of the treated lesions were BTK. These investigators showed a procedural success rate of 88% with mean wound area reduction of 89% at 6 months and a 69% limb salvage rate [39]. In the Laser-Assisted Angioplasty for Critical Limb Ischemia (LACI) trial, 423 lesions were treated with LA and 41% were specifically BTK. The overall success rate was 86% and the limb salvage rate was 93% at 6 months [40]. Finally, in 2013 the Laser in infra-popliteal and popliteal stenosis (LIPS) retrospective review, analyzed LA vs PTA for IPOD. The study showed that LA plus PTA had a 5 times greater likelihood of improvement in the IPA lesion severity score compared to PTA alone [41].

### 1.8. Treatment

Once vessel preparation has been optimized, treatment can be performed with several treatment options today. These include PTA, drug coated balloons (DCBs), and drug eluting stents (DESs).

### 1.9. Percutaneous Transluminal Angioplasty (PTA)

The gold standard for treatment of IPA disease is PTA. A meta-analysis by Mustapha et al. included 52 studies encompassing 6769 patients with 9399 IPA lesions which were included in the analysis. Technical success was 91.1% and the incidence of flow-limiting dissections and bailout stenting was 5.6% and 9.1%, respectively. Outcomes at 1 year were primary patency, 63.1%; repeat revascularization, 18.2%; major amputation, 14.9%; and all-cause mortality, 15.1%. They note significant heterogeneity and publication bias for most of the PTA outcomes. Overall their analysis showed that the use of PTA as a primary treatment for patients with IPOD had suboptimal procedural and 1-year clinical outcomes with associated renal disease resulting in increased risk of failed patency and increased incidence of revascularization [42].

### 1.10. Drug Coated Balloon (DCB)

A DCB is a balloon that is coated with paclitaxel, an anti-cancer drug, and a carrier molecule or excipient. This excipient allows the drug to come off the balloon to be delivered into the vessel wall where it resides long enough to inhibit smooth muscles cells and impact neo-intimal proliferation. Key advantages of DCBs are that the operator does not need to worry about malposition, as in stent placement, and future vascular surgical interventions are not limited if needed. DCBs in this category include the In.PACT Admiral (Medtronic), Lutonix (BD Peripheral Vascular Inc.), and Stellarex (Phillips) (Table 1).

Each DCB differs in drug density and drug-coating [43]. Although data for DCBs show superiority in reducing restenosis events in the femoropopliteal region, currently no DCB has an FDA indication for IPOD in the United States. However, some data can be gleaned about their effectiveness in the BTK region from various trials.

To date the use of DCBs in the BTK region have been mixed. In a few randomized control trials (In.PACT DEEP, BIOLUX P-II), the results have not been substantial [44,45,46]. The more recent Lutonix BTK trial at 6-month follow-up did show a statistically significant primary patency, freedom from above-ankle amputation, and clinically driven (CD) TLR of Lutonix DCB over PTA. However, clinical and functional outcomes were similar [47].

A meta-analysis of RCTs by Katsanos et al. investigated DCBs vs PTA for the treatment of IPOD in CLI patients [48]. The primary safety and efficacy endpoint was amputation-free survival (AFS) defined as freedom from all-cause death and major amputation. TLR constituted a secondary efficacy endpoint. 8 randomized controlled trials with 1420 patients (97% CLI) were analyzed with up to 1-year follow-up. AFS was significantly worse in the case of DCB (13.7% crude risk of death or limb loss compared to 9.4% with PTA (*p* = 0.008). TLR was significantly reduced in the case of DCB (11.8% crude risk of TLR versus 25.6% with PTA (*p* = 0.004). The harm signal was evident when examining the high-dose (3.0–3.5 mg/mm^2^) DCBs, but attenuated below significance in the case of low-dose (2.0 mg/mm^2^) DCBs. Actual causes remain largely unknown, but non-target paclitaxel embolization was indicated as a plausible mechanism [48].

### 1.11. Drug Eluting Stent (DES)

To date coronary DESs are often used to treat IPA disease off label. Multiple studies have shown increased effectiveness and promising clinical outcomes for treatment of selected focal lesions [49]. Varcoe et al. performed a meta-analysis of randomized controlled trials comparing DES with conventional treatment for symptomatic IPA disease. The primary endpoint was primary patency and secondary endpoints were freedom from TLR, major amputation, sustained RC improvement and mortality. In their review, a total of 7 trials enrolling 801 randomly assigned patients (392 DES, 409 control) were included. Primary patency at 6 months was 73.6% in the DES group compared with 51.3% in the control group. Similar results were observed at 12-months, with a primary patency of 75.8% in the DES group and 47.9% in the control group. The use of DES significantly reduced the risk of TLR compared to standard EVT at 6-months and 12-months. At 6-months 94.0% of the patients treated with DES were free from TLR and 84.7% in the control group. At 12-months 85.0% of the patients treated with DES were free from TLR and 72.1% in the control group. There was a trend towards lower major amputation rates with DES in patients with CLI. As with previous reviews, there was no difference in overall survival between DES and the control groups [50].

Katsanos et al. performed a meta-analysis of RCTs comparing the effectiveness of PTA, bare metal stents (BMS), DCBs, and DESs for the treatment of IPOD. In this analysis 16 RCTs comprising 1805 patients with 1-year median follow-up were analyzed. There was high quality of evidence showing infrapopliteal DES significantly reduced restenosis compared with BMS and PTA. Likewise, DES significantly reduced TLR compared with PTA and BMS. DCBs (paclitaxel coated) also had a reduced TLR compared with PTA and BMS but quality of evidence was low to moderate. PTA had a lower TLR than BMS with high quality of evidence. DES was the only treatment that significantly reduced limb amputations compared with PTA, DCB, or BMS with moderate to high quality of evidence. DES also significantly improved wound healing compared with PTA or BMS with high quality of evidence. Results were stable without significant publication bias or inconsistency. Overall, they found that infrapopliteal DES were associated with significantly lower rates of restenosis, TLR, and amputations and improved wound healing compared to PTA and BMS. DES also significantly reduced amputations compared with DCB [49].

Despite excellent data, there are no dedicated DESs for BTK disease. Coronary DESs are traditionally used but they are used off label. Boston Scientific Inc recently began studying a dedicated DES for IPOD with the start of their The DES BTK Vascular Stent System vs PTA in Subjects With Critical Limb Ischemia (SAVAL) trial. It is a global, prospective, randomized, multicenter trial designed to assess the safety and efficacy of the SAVAL BTK DES System compared to PTA in treating patients with CLI and IPOD. The study will include approximately 201 patients at 50 sites in the US, Europe, and Japan. Final results are not available currently [51].

### 1.12. TACK Endovascular System

Despite optimal VP and treatment, complications can arise including dissections. Giannopoulos et al. showed that dissections occur in 6.4–30.7% of cases after IPA PTA [52]. Prolonged low-pressure PTA and stent placement are often used, but stent placement including DES is off label for IPOD.

**Figure 9:**
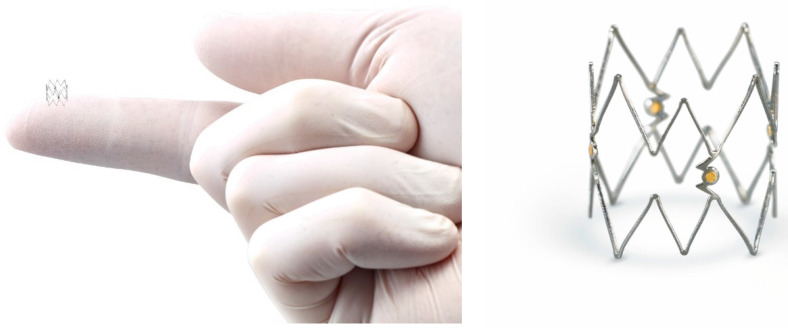
BTK Tack Endovascular System. Courtesy of Intact Vascular Inc.

Recently the 4 FR Tack Endovascular System (Intact Vascular Inc, Wayne, PA, USA) is a US Food and Drug Administration approved minimal metal implant for dissection repair in the tibial and/or peroneal arteries (Figure 9).

It is designed to repair dissections following PTA. Preloaded with 4 self-expanding nitinol devices for BTK interventions, the Tack Endovascular System can be deployed to treat multiple dissections using a single catheter and leaving behind >70% less metal than stents [53]. TOBA II BTK is a prospective, single-arm, pivotal investigational device exemption (IDE) study looking at the Tack Endovascular System in 233 patients at 41 US and international sites. The trial looked at patients with CLI and angiographic evidence of a BTK dissection after PTA requiring repair in the tibial and/or peroneal arteries ranging in diameter from 1.5 mm to 4.5 mm. It is the first trial to enroll 100% dissected vessels. The study met all its primary endpoints with 100% acute dissection resolution and 73.8% of wounds healed or improved at six months. Results demonstrated 95.7% K-M AFS and 87.3% K-M target lesion patency with significant improvement in toe-brachial index (TBI) and 92.0% K-M freedom from CD reintervention [53].

## 2. Conclusions

In general, CLI patients with associated IPOD are complex and technically challenging. In addition, there are limited bailout options when dealing with BTK complications. As a result, those treating patients with CLI and IPOD must be proficient in the various devices available for vessel preparation and treatment in order to optimize patency rates, symptom resolution, healing of wounds, and minimize complications.

## Figures and Tables

**Table 1:** Food and Drug Administration (FDA) Approved Drug Coated Balloons. Non.

Manufacturer	DCB	Drug	Dose (Micrograms/mm^2^)
BD Bard	Lutonix	Paclitaxel	2.0
Medtronic	In.PACT	Paclitaxel	3.5
Philips	Stellarex	Paclitaxel	2.0
